# Numerical Modeling of Particle Dynamics Inside a Dry Powder Inhaler

**DOI:** 10.3390/pharmaceutics14122591

**Published:** 2022-11-24

**Authors:** Tijana Šušteršič, Aleksandar Bodić, Jelisaveta Ignjatović, Sandra Cvijić, Svetlana Ibrić, Nenad Filipović

**Affiliations:** 1 Faculty of Engineering, University of Kragujevac, Sestre Janjić 6, 34000 Kragujevac, Serbia; 2 Bioengineering Research and Development Center (BioIRC), Prvoslava Stojanovica 6, 34000 Kragujevac, Serbia; 3 Department of Pharmaceutical Technology and Cosmetology, Faculty of Pharmacy, University of Belgrade, Vojvode Stepe 450, 11000 Belgrade, Serbia

**Keywords:** dry powders inhalers (DPI), computational fluid dynamics (CFD), discrete phase model (DPM), particle sticking, DPI efficiency

## Abstract

The development of novel dry powders for dry powder inhalers (DPIs) requires the in vitro assessment of DPI aerodynamic performance. As a potential complementary method, in silico numerical simulations can provide additional information about the mechanisms that guide the particles and their behavior inside DPIs. The aim of this study was to apply computational fluid dynamics (CFDs) coupled with a discrete phase model (DPM) to describe the forces and particle trajectories inside the RS01^®^ as a model DPI device. The methodology included standard fluid flow equations but also additional equations for the particle sticking mechanism, as well as particle behavior after contacting the DPI wall surface, including the particle detachment process. The results show that the coefficient of restitution between the particle and the impact surface does not have a high impact on the results, meaning that all tested combinations gave similar output efficiencies and particle behaviors. No sliding or rolling mechanisms were observed for the particle detachment process, meaning that simple bouncing off or deposition particle behavior is present inside DPIs. The developed methodology can serve as a basis for the additional understanding of the particles’ behavior inside DPIs, which is not possible using only in vitro experiments; this implies the possibility of increasing the efficiency of DPIs.

## 1. Introduction

Drug delivery via the pulmonary route is an important area that has been actively researched in recent years. Many inhalation devices have been developed for these purposes, including dry powder inhalers (DPI). DPIs are devices used to deliver a dry powder formulation of a drug to the lungs for the purpose of treating a variety of respiratory diseases [1]. In order to be successfully delivered and deposited in the lungs, the particles should have an aerodynamic diameter of 0.5 µm to 5 µm, while those with an aerodynamic diameter of less than 3 µm have the chance of reaching the respiratory zone [2]. The particles of such a small diameter generated by the inhaler are very cohesive and can either form drug-only agglomerates or can be attached to larger carrier particles [3,4]. In order for the particles to be effectively transported and deposited in the lungs, processes of particle dispersion and deaggregation are required. These processes occur due to the action of the fluid on the particles and wall–particle impact. Therefore, particle detachment from the inhaler’s wall (surface) depends on the established flow during inhalation and the geometry of the device [5]. Finally, drug particles which are small enough are transported and deposited in the alveolar region of the lungs.

The biggest problem with drug delivery using DPI devices is that a large proportion of the particles stay deposited inside the device. Therefore, a very small percentage of the drug (less than 30%) reaches the target pulmonary regions [6]. In addition, it is necessary to achieve good control over the process of deaggregation, i.e., the separation of drug particles from carrier particles in order to ensure the inhalation of an equal drug dose with each subsequent use of the inhaler [7]. As experimental investigations in this area encounter many practical challenges, the application of computational modeling of fluid flow and particle dynamics is becoming more frequent. For the purpose of efficient device design, computational fluid dynamics (CFDs) are used to determine the airflow pattern and turbulence levels, as well as to model the transport of the particles through the device and their interaction with the inhaler wall [8]. 

### Related Work

Previous research in this area has mainly been related to the determination of the flow structure and simulation of the movement of particles through the device. The simulation of airflow through an inhaler traditionally refers to the numerical solution of Reynolds-Averaged Navier–Stokes (RANS) equations that are associated with an adequate turbulence model [9,10]. Because some turbulent models have limited application in modeling turbulent swirling flows in the inhaler, and since time averaging has many disadvantages, more accurate data on fluid flow can be obtained by applying large eddy simulations (LESs) [11,12]. In the study by Milenkovic et al. [13], a comparison of the results of the CFD simulation using different turbulence models with LES and experimental data showed that the most accurate results are obtained by the k-w SST turbulence model. The aforementioned study also showed that LES has high computational requirements, and therefore is not widely applied in practice. In a later study, using the same DPI device geometry, Milenkovic et al. [14] modeled dynamic flow instead of stationary flow. The particle deposition results obtained by the dynamic CFD models were shown to match well with the experimental in vitro results. Also, the fine particle fraction (FPF) results obtained by the dynamic CFD models matched the experimental results, while the steady flow simulations could not determine the correct FPF values.

The development of CFD analysis created the possibility of modeling the transport and deposition of aerosol particles in addition to simulations of turbulent and laminar airflow through DPI devices [15]. There are several fluid-particle dynamic (CF-PD) models for calculating air–drug mixture dynamics, which include methods such as the discrete phase model (DPM), mixture models, and discrete element methods (DEM), as well as dense dispersed phase models (DDPMs) [16]. Coupled CFD-DPM is used to analyze various types of commercial DPI devices, which have been the subject of research in many studies [13,17,18,19]. Using the Lagrangian approach with one-way coupling, Sommerfeld et al. [9] investigated the influence of different fluid forces and wall collision modeling on particle behavior. In their study, it was shown that wall collisions have a significant influence on the movement of particles from which the dispersion of the particles occurs. It was also shown that, with an increase in particle size, the frequency of wall-impact increases, but the wall-impact velocity decreases. Similar conclusions were reached in the study of Donovan et al. [20], where the influence of particle physical properties and device type on aerosol performance was considered. Their study showed that, for both types of inhalers considered, the wall-impact rate increases with increasing particle diameter. 

The influence of inhaler geometry on flow structure and particle motion has been investigated in several studies [21,22,23]. In these studies, it was concluded that mouthpiece length, grid, and inlet size significantly affect the performance of the inhaler. The optimization of inhaler performance by modifying its geometry has been investigated by Milenkovic et al. [24]. This study shows that simple changes in device geometry can significantly improve the generated flow and can increase FPF, i.e., improve the efficiency of the inhaler. An investigation of the powder dispersion mechanism using CFD and DEM was presented by Tong et al. [7]. It was shown that the impact of particles on the inhaler wall in the area of the grid plays a key role in the increase in FPF. 

Although several papers [9,13,20] investigated the behavior of particles inside the DPIs and particle wall collision, there are no published studies investigating whether a particle rebounds after impacting a wall. Our previous publication [25] compares in vitro and in silico methods for DPI aerodynamic characterization, with the goal of comparing the results of the CFD-DPM simulations with the results of three in vitro methods for the DPI aerodynamic assessment of solid lipid microparticles. In the current study, we focus on the development of CFD-DPM methods to investigate the particle detachment process (sliding and rolling), including the fluid dynamic interaction between the flow and the particles stuck to the wall, by defining equations to describe the sticking and rebounding (sliding and rolling) mechanisms. The main contribution of this paper is to investigate the underlying mechanisms of particle behavior inside DPIs using numerical simulations. The geometry was created using RS01^®^ as a model DPI device, after which coupled CFD and DPM computational simulations were performed to determine both the fluid flow and particle behavior. The simulation results were compared with the results from the literature, specifically in terms of the total particle deposition presented in previous publications [26,27] based on in vitro experiments and the deposition obtained in the study of Milenkovic et al. [13], which came from numerical simulations. 

## 2. Materials and Methods

### 2.1. Geometry and Meshing

**Figure 1:**
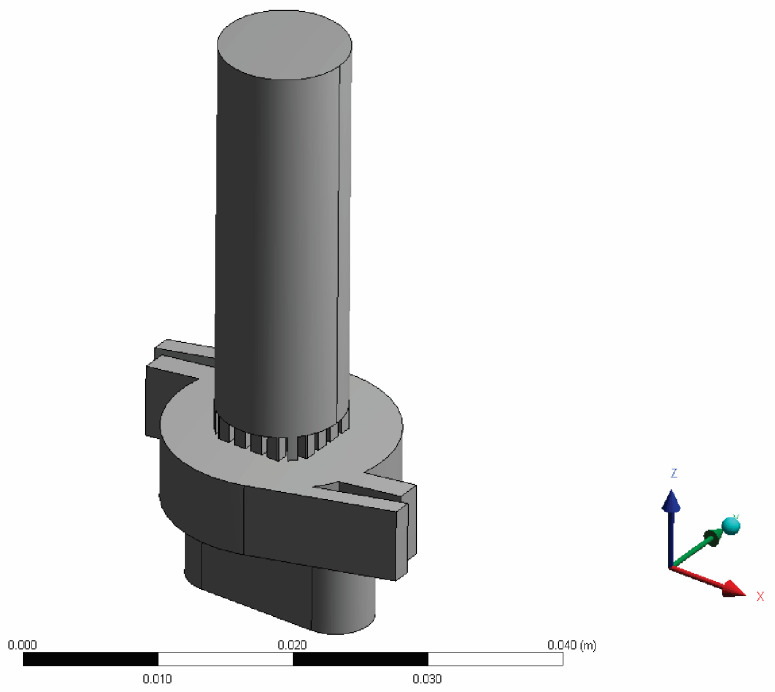
Device geometry; isometric view.

The device geometry was created based on the real DPI device. The DPI device considered in this paper was an RS01^®^ inhaler, which was used in our previous research [25]. The inhaler geometry was obtained using commercial CAD software designed for these purposes (i.e., CATIA version 5, Dassault Systems, France), and is shown in Figure 1. 

**Figure 2:**
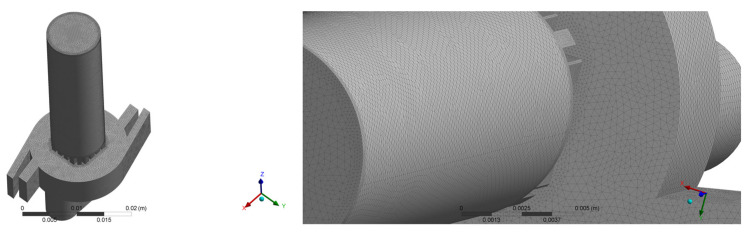
Meshing of the DPI geometry.

Based on the created DPI geometry, an optimal finite volume mesh was generated using Ansys fluent meshing (Figure 2).

Fluent meshing was used to evaluate the mesh’s quality, and the mesh was then used in Fluent to simulate airflow. Initially, the common planes and edges were meshed, followed by the creation of a volume mesh. Refinements to the planes, edges, and corners corresponding to the walls were made during the meshing process. Volumetric meshes were altered in areas where considerable velocity gradients were expected. It was discovered that at least seven grid points in the near wall region, y + 2.5, are required. To meet this condition, the computational grids in this study were increased in the near-wall region. Computational meshes had dimensions ranging from 2 × 10^5^ to 2 × 10^7^ and were composed of tetrahedral cells, modeled based on the data from Milenkovic [28]. The cells had a maximum skewness of 0.85. To determine mesh independence, total particle depositions for six different meshes (about 2 × 10^5^, 5 × 10^5^, 1 × 10^6^, 2 × 10^6^, 5 × 10^6^, and 1 × 10^7^) were compared to 100% deposition assumptions. According to these simulations, the 2 × 10^6^ mesh gave enough resolution to obtain realistic particle simulation results. As a consequence, the mesh with the total number of nodes 349,460 and the number of cells 1,930,248 (≈2 × 10^6^) was employed to get the results presented in this study.

The boundary conditions were set on the previously defined regions of the model, i.e., the inlet, outlet, and inhaler’s wall. This included defining wall surfaces and the inlet and outlet pressures. Fluid flow through the inhaler is driven by the difference in inlet and outlet pressures. In order to achieve a peak inspiratory flow rate (PIFR) of 60 l/min, which is considered optimal for powder deaggregation into fine particles that can reach the lungs [29], a pressure drop of 2800 Pa was set. Firstly, the airflow through the inhaler was simulated, and after the steady flow was fully formed, particles inside the inhaler were released. A steady-state solution was considered converged in cases where residuals were less than 10^−4^. According to Milenkovic et al. [13] instantaneous volumetric flow rate increases rapidly and reaches PIFR, i.e., maximum value. Therefore, a steady-state airflow may be considered as a close approximation to dynamic airflow developed in DPI, because for most of the inhalation process duration the instantaneous flow rate is approximately equal to the PIFR. Consequently, steady-state airflow was considered in this paper. Particles were released from a height of 12.5 mm, which corresponds to the real position of the drug capsule inside the device. The velocity of particles was set to correspond to the fluid velocity in that region of the inhaler. 

In order to establish the particle number from an independent study, the model was tested by performing numerical simulations with a different number of particles. Simulations were performed with 100, 500, and 5000 injected particles to achieve the consistency of the solution, despite the number of particles. As the behavior of the model was identical for all three models, a number of 500 particles was adopted in order to reduce the computational time and resources and also to obtain adequate solutions for presentation and interpretation. 

Numerical simulations were carried out using processing hardware that includes 32 GB of RAM and an Intel(R) Core (TM) i5-4590 CPU running at 3.30 GHz (4 CPUs), with computational time ranging from 3 to 4 h. 

### 2.2. CFD Modeling

In the CFD model, fluid motion is specified by time-averaged conservation of mass and momentum equations, known as Navier–Stokes equations. The turbulent flow through the inhaler may be described by connecting these equations with an appropriate turbulence model. Navier–Stokes time-averaged equations are named Reynolds Averaged Navier–Stokes (RANS) equations, and are defined as follows:

(1) $$\frac{\partial {\bar{u}}_{i}}{\partial t}+\frac{\partial }{\partial x_{j}}\left({\bar{u}}_{j}{\bar{u}}_{i}\right)=-\frac{\partial \bar{p}}{\partial x_{i}}+\frac{\partial }{\partial x_{j}}\left[\upsilon \left(\frac{\partial {\bar{u}}_{i}}{\partial x_{j}}+\frac{\partial {\bar{u}}_{j}}{\partial x_{i}}-\bar{{u^{\prime }}_{i}{u^{\prime }}_{j}}\right)\right]$$

where *u* denotes velocity, *p* represents the pressure divided by density $p=\frac{P}{\rho }$, and $\upsilon =\frac{\mu }{\rho }$ represents the kinematic viscosity. As a consequence of the nonlinearity of the Navier–Stokes equations, the term $\bar{{u^{\prime }}_{i}{u^{\prime }}_{j}}$ appears, which consists exclusively of fluctuating values. This term represents a symmetric nonlinear second-order tensor with six unknown variables, which is called Reynolds stress tensor. By introducing six new unknown variables, the system has more unknown variables than equations. For this reason, this tensor is modified to represent the function of the averaged values exclusively, and the concept of turbulent dynamic viscosity, *μ_t_*, is introduced:

(2) $$-\rho \bar{{u^{\prime }}_{i}{u^{\prime }}_{j}}=\mu_{t}\left(\frac{\partial {\bar{u}}_{i}}{\partial x_{j}}+\frac{\partial {\bar{u}}_{j}}{\partial x_{i}}-\frac{2}{3}\frac{\partial {\bar{u}}_{k}}{\partial x_{k}}\delta_{ij}\right)-\frac{2}{3}k\delta_{ij}$$

where $\delta_{ij}$ represents the Kronecker delta, and *k* is the specific turbulent energy. Assuming an incompressible flow:

(3) $$\frac{\partial {\bar{u}}_{k}}{\partial x_{k}}=0$$

The Reynolds tensor can be written via the turbulent dynamic viscosity for incompressible flow in the following form:

(4) $$-\rho \bar{{u^{\prime }}_{i}{u^{\prime }}_{j}}=\mu_{t}\left(\frac{\partial {\bar{u}}_{i}}{\partial x_{j}}+\frac{\partial {\bar{u}}_{j}}{\partial x_{i}}\right)-\frac{2}{3}k\delta_{ij}$$

Depending on the approach by which the turbulent dynamic viscosity is defined, different RANS turbulent models are developed. Most often, turbulent flow properties are represented by two-equation turbulence models *k-ω* and *k-ε*, where *ω* denotes the specific turbulence dissipation rate and *ε* denotes the dissipation rate of turbulent kinetic energy. In this paper, the shear stress transport (SST) *k-ω* turbulent model was used, which according to Milenkovic et al. [13] gives the most similar results to large eddy simulations (LES) results, as well as experimental results for the pressure drop in the DPI. The *k-ω* SST model is a hybrid model which combines the *k-ω* and the *k-ε* models in order to eliminate their disadvantages. This model uses the *k-ω* in regions close to the wall, while in free stream, it switches to the *k-ε* model. The transport equation for turbulent kinetic energy is given in the following form:

(5) $$\frac{\delta k}{\partial t}+U_{j}\frac{\delta k}{\partial x_{j}}=P_{k}-\beta^{*}k\omega +\frac{\delta }{\partial x_{j}}\left[\left(\upsilon +\sigma_{k}\upsilon_{t}\right)\frac{\delta k}{\partial x_{j}}\right]$$

The transport equation for dissipation rate of turbulent kinetic energy is given by Equation (6):

(6) $$\frac{\partial \omega }{\partial t}+U_{j}\frac{\partial \omega }{\partial x_{j}}=\alpha S^{2}-\beta \omega^{2}+\frac{\partial }{\partial x_{j}}\left[\left(\upsilon +\sigma_{\omega }\upsilon_{t}\right)\frac{\partial \omega }{\partial x_{j}}\right]+2\left(1-F_{1}\right)\sigma_{\omega 2}\frac{1}{\omega }\frac{\partial k}{\partial x_{i}}\frac{\partial \omega }{\partial x_{i}}$$

Turbulent kinematic viscosity is calculated using the following equation:

(7) $$\upsilon_{t}=\frac{a_{1}k}{\max(a_{1}\omega ,SF_{2})}$$

where *a*_1_ represents an empirically determined constant, *S* is defined by the strain rate tensor *S_ij_* and functions *F*_1_ and *F*_2_ give the connection between the *k-ω* and *k-ε* models.

### 2.3. Particle Sticking Process

The particle sticking mechanism is influenced by a number of factors, including particle size, angle of impact, velocity, and the particle and the contact wall surface properties. It is frequently the result of one or more of the following mechanisms: the formation of van der Waals and electrostatic forces in dry conditions and liquid bridge forces in wet conditions [30]. The van der Waals forces are caused by molecular interactions between two surfaces, meaning there is a particle and a wall. If the arriving particles in the gas or fluid stream are electrically charged, electrostatic force contributes to the sticking process. The formation of a liquid bridge between the particle and the touch surface causes the intensity of the liquid bridge to increase. The liquid bridge is made up of an isothermal mass of liquid held together by surface tension between two bodies in contact [31]. Here, dry conditions were assumed; therefore, no liquid bridge existed. Soltani and Ahmadi [32] carried out a study of the adhesion mechanisms and stated that van der Waals force is the main contributor to particle adhesion under dry conditions. Soltani and Ahmadi [32] calculated the sticking power using the sample scale and the 12 properties of the object and surface material. The sticking power, $F_{st}$, is defined by Soltani and Ahmadi [32] as the following:

(8) $$F_{st}=\frac{3}{4}\pi W_{A}d_{p}$$

where $W_{A}$ is the work of adhesion and $d_{p}$ is the particle diameter. The work of adhesion for silicon–silicon surfaces that are in contact is available from the literature, which has been experimentally determined by Soltani and Ahmadi [32] and is equal to 38.9 × 10^−3^ J/m^2^.

Dahneke [33] established the criterion for surface-sticking particles. He investigated the impact velocity of particles on the rebound velocity of spherical shape particles. According to him, when the normal impact velocity ($v_{n}$) decreases, the importance of the sticking force rises, resulting in a decrease in rebound velocity. There is no rebounding of particles under the critical value of the normal impact velocity, and the particles adhere to the surface. This velocity is known as the capture velocity. Brach and Dunn [34] computed the capture velocity based on experimental data using a mathematical model for the impact and adherence of spherical particles. The capture velocity $v_{cr}$ is provided as follows:

(9) $$v_{cr}={\left[\frac{2E}{d_{p}}\right]}^{\frac{10}{7}}$$

where:

(10) $$E=0.51{\left[\frac{5\pi^{2}(k_{1}+k_{2})}{4\rho_{p}{}^{3/2}}\right]}^{\frac{2}{5}}$$

is the El Batch parameter, also defined in the paper by Alden et al. [35]. The terms $k_{1}$ and $k_{2}$ are defined by the Equations (11) and (12):

(11) $$k_{1}=\left(\frac{1-v_{s}{}^{2}}{\pi E_{s}}\right)$$

(12) $$k_{2}=\left(\frac{1-v_{p}{}^{2}}{\pi E_{p}}\right)$$

$E_{s}$ and $E_{p}$ are the surface and particle materials’ Young’s moduli, respectively, whereas $v_{s}$ and $v_{p}$ are the surface and particle materials’ Poisson’s ratios, respectively. A particle with a normal impact velocity larger than the critical velocity $v_{n}>v_{cr}$ will bounce off the surface when it comes into contact with it. This signifies that the deposition will take place if the previous condition is satisfied. Table 1 defines all of the other constants. 

### 2.4. Particle Detachment Process

**Figure 3:**
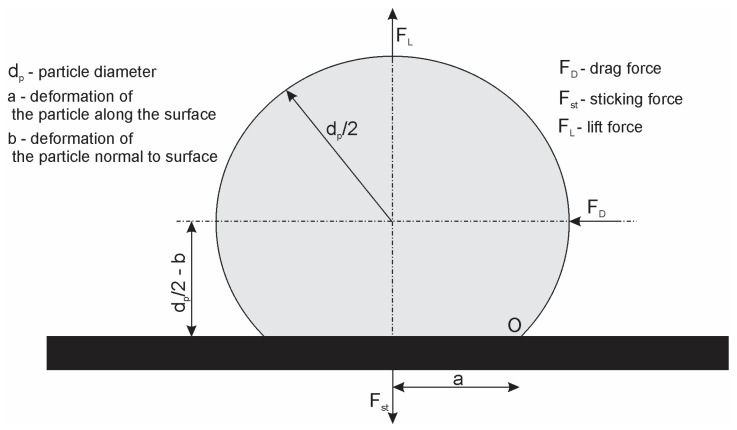
Forces acting on a deformed particle stuck to a wall.

When the fluid forces are strong enough to overcome the particle adhesion forces, the deposited particles are released and resuspended. Soltani and Ahmadi [32] looked at several particle detachment methods. Rolling and sliding can both influence particle detachment; however, rolling is the most likely process for spherical particles. Figure 3 depicts an overview of investigated particle forces.

If the particle bounces and continues along the trajectory in the flow, its rebound velocity will be decreased, which is determined by the coefficient of restitution (COR) between the particle and the surface. Because COR values from in vitro studies were not available, the simulations explored a variety of various combinations of normal and tangential COR. We examined all combinations for the values 0.2, 0.25, 0.5, 0.75, and 0.85 [36].

#### 2.4.1. Detachment by Rolling

The particle begins to roll and detaches in the case where the moment produced by the fluid forces at a certain stage on the particle-wall-interaction interface is greater than the moment induced by the adhesion force. The fluid and adhesion forces acting on a particle are shown in Figure 3. When the following condition (summing the moments around the point O) is met, the stuck particles will be released from the surface.

(13) $$F_{D}\left(\frac{d_{p}}{2}-b\right)+F_{L}a\geq F_{st}a$$

In Equation (13), $F_{D}$ is the drag force, $F_{L}$ is the lift force, a represents the distance along the surface from the particle center to point O (deformation of the particle along the surface), and b is the deformation of the particle normal to the surface. According to Soltani and Ahmadi [32], the influence of lift force on detachment is small when compared to drag force. In the case of elastic particle adhesion,b is small in comparison to the particle diameter $d_{p}$, and may thus be ignored. As a result, the particle separation by the rolling condition is simplified to the following equation:

(14) $$F_{D}\left(\frac{d_{p}}{2}\right)\geq F_{st}a$$

The distance a along the surface to point O from the particle center representing the deformation along the surface is given by Soltani and Ahmadi [32] as:

(15) $$a=\sqrt[{3}]{\frac{3\pi }{2}\frac{W_{A}d_{p}{}^{2}}{K_{C}}}$$

where *K_C_*, defined by Equation (16), is the composite Young’s modulus:

(16) $$K_{C}=\frac{4}{3}{\left[\frac{\left(1-v_{s}{}^{2}\right)}{E_{s}}+\frac{\left(1-v_{p}{}^{2}\right)}{E_{p}}\right]}^{-1}$$

#### 2.4.2. Detachment by Sliding

Wang [37] studied the effects of initial motion on particle detachment from surfaces and established a sliding particle detachment condition. When the fluid drag force is high enough to cause the particle to move, a particle will detach from the surface, which occurs in case the following condition is met:

(17) $$F_{D}\geq k_{s}F_{st}$$

Here, $k_{s}$ is the coefficient of static friction between the particle and the wall.

Limiting Conditions for Detachment by Rolling and Sliding

The drag force on a spherical particle is defined by equation:

(18) $$F_{D}=\frac{1}{2}C_{D}\rho V^{2}\left(\frac{\pi d_{p}}{4}\right)\left(\frac{f}{C_{u}}\right)$$

where the drag coefficient is given by the equation:

(19) CD=24Rep

Reynolds number is given by Equation (20).

(20) Rep=dpρVμ

Soltani and Ahmadi [32] provided a correction factor, f, for the near-wall effect, and the value is shown in Table 1. Table 1 also includes the value of the Cunningham correction factor, $C_{u}$, for spherical particles. $V=\sqrt{u^{2}+v_{n}{}^{2}}$ is the fluid velocity at the particle’s center, where u and $\upsilon_{n}$ represent the fluid velocity components parallel to and normal to the wall, respectively. Because the flow runs parallel to the wall $\upsilon_{n}=0$, we may conclude that V=u. It is possible to define a particle in the viscous sublayer as follows:

(21) $$V=\frac{\rho }{\mu }\frac{d_{p}}{2}{\left(u^{*}\right)}^{2}$$

which leads to the equation for $F_{D}$:

(22) $$F_{D}=\frac{5.1\pi }{2}d_{p}{}^{2}\rho u^{{*}^{2}}$$

where $u^{*}$ is the wall shear velocity. 

The limiting condition, $u^{*}$ is defined as $u_{R}{}^{*}$ for rolling and $u_{s}{}^{*}$ for sliding. These values are then called *critical wall shear velocities*. Substituting the formula for drag force in the rolling limiting condition (Equation (14)) produces the rolling critical wall shear velocity:

(23) $$u_{R}{}^{*}=\sqrt{\left(\frac{1}{\rho }\right){\left(\frac{1}{K_{C}}\right)}^{1/3}{\left(\frac{W_{A}}{d_{p}}\right)}^{4/3}}$$

Substituting the expression for drag force in the sliding limiting condition (17) yields a critical wall shear velocity for sliding:

(24) $$u_{s}{}^{*}=0.5\sqrt{\frac{k_{s}W_{A}}{\rho_{p}d_{p}}}$$

In order to detach and resuspend a particle in the flow, the wall friction velocity $u^{*}$ must be greater than the critical wall shear velocities for both rolling and sliding circumstances, which means:

(25) $$u^{*}\geq u_{R}{}^{*}$$

(26) $$u^{*}\geq u_{s}{}^{*}$$

In the context of the finite element method (FEM), $u^{*}$ is given by:

(27) $$u^{*}=\frac{\mu y^{+}}{\rho_{M}d_{s}}$$

where μ is the dynamic viscosity of fluid, $\rho_{M}$ is the mixture density, and $d_{s}$ is the distance of the first grid point from the wall.

### 2.5. User Defined Functions (UDF)

**Figure 4:**
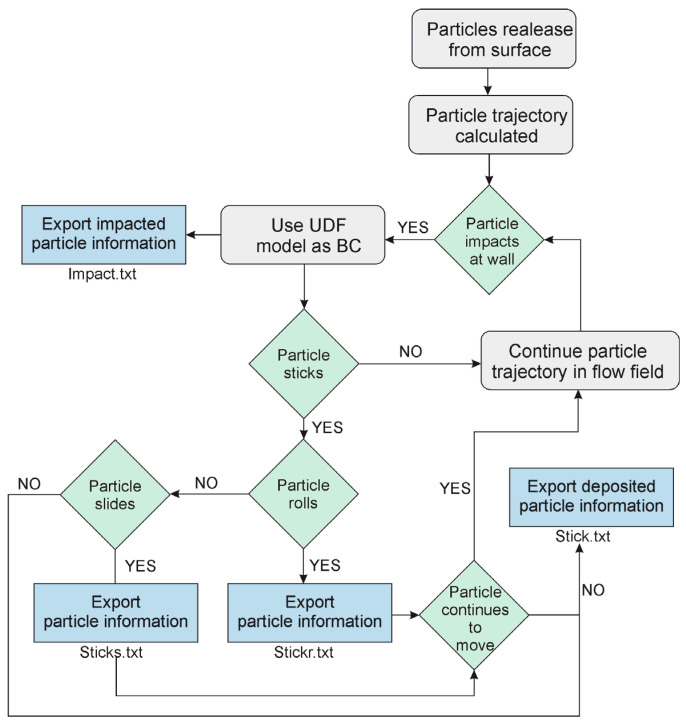
Description of particle behavior—proposed methodology to track particle deposition and detachment.

Based on the described methodology and equations, the workflow is illustrated in Figure 4. The known input parameters for the model are material characteristics such as material properties, Young’s modulus, Poisson’s ratio, the density and viscosity of the DPI wall surface and particles, as well as the coefficient of static friction.

The boundary conditions at the inhaler’s walls are described by the user-defined function (UDF), which simulates particle sticking and detachment mechanisms by applying the relations described in the previous section. The UDF calculates the critical velocity of a particle using Equation (5) and, comparing it with the normal velocity of a particle, determines whether the particle sticks to the wall or bounces. If the particle does stick to the wall, the UDF then applies Equations (14) and (18) to determine if the particle will bounce back into the airflow by rolling, or via Equations (17) and (19) to determine if the particle will bounce back by sliding. If none of the conditions are met, the particle remains stuck to the device’s wall. After the calculation is completed, the UDF writes several files containing data on the particles that are stuck, detached by rolling, and detached by sliding.

## 3. Results

Modeling particle dynamics within DPI devices involves airflow, powder dispersion, aggregate breakage, and particle deposition in the inhaler and is, therefore, rather complicated. The coupled CFD-DPM model of a DPI considered in this paper has been used to determine dynamic flow, particle deposition in the inhaler, and FPF. Key outputs of numerical simulations are the emitted flow, FPF, and the total number of deposited particles in the inhaler.

**Figure 5:**
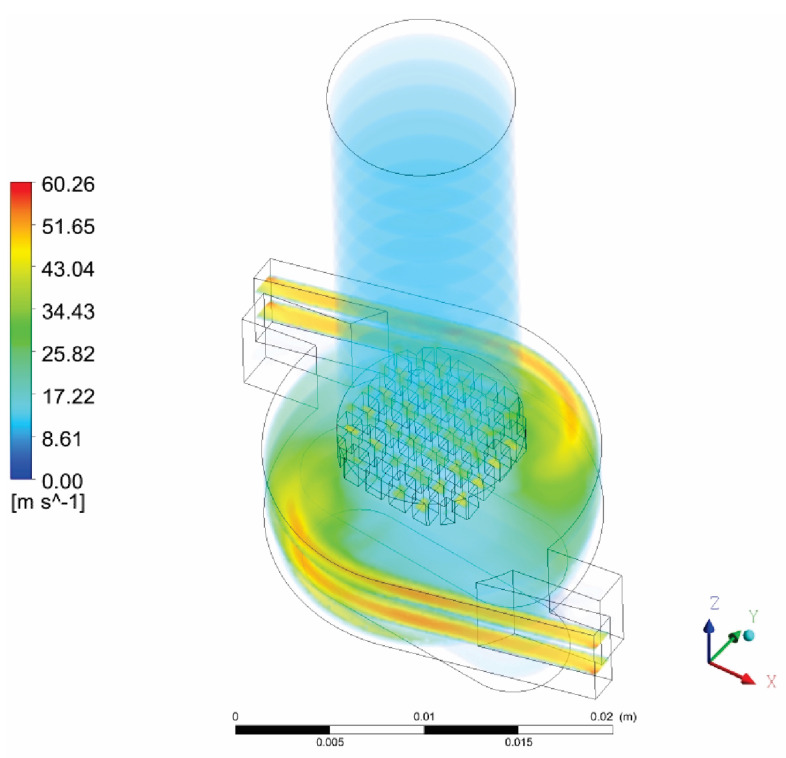
Fluid velocity magnitude in 3D model.

The results for airflow through the inhaler are shown in Figure 5 in the form of fluid velocity magnitude. Figure 5 shows that larger eddies occur in the middle chamber of the inhaler. Higher values of fluid velocity magnitude occur from the inlets to the middle chamber and in the grid zone of the inhaler. At the outlet, it is noticed that the velocity has a value that corresponds to the flow rate of 60 l/min, i.e., approximately 12 m/s. 

**Figure 6:**
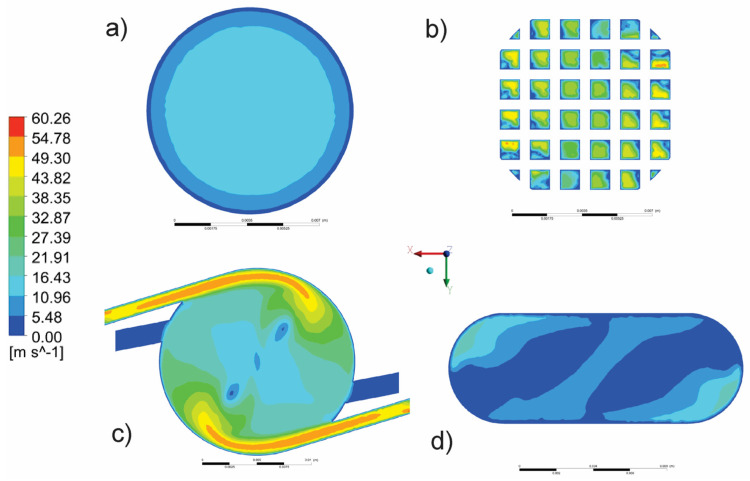
Fluid velocity magnitude in characteristic cross-sections: (**a**) outlet, (**b**) grid zone, (**c**) central chamber, and (**d**) capsule chamber.

In addition, Figure 6 shows the velocity magnitude field in the outlet (a), as well as in the characteristic cross-sections, such as the horizontal cross-section in the grid zone (b), central chamber of the inhaler (c), and capsule chamber (d).

After a steady-state solution for airflow was established, the particles were inserted into the inhaler. The height from which the particles were released was defined to correspond to the real position of the capsule within the DPI, i.e., 12.5 mm from the bottom of the device. The initial velocity value of the particles was set to correspond to the fluid velocity at that height.

Because the velocity at which the particle will eventually bounce off of the wall depends on the COR, several numerical simulations were performed for different values of the normal and tangential COR, as shown in Table 2. 

**Figure 7:**
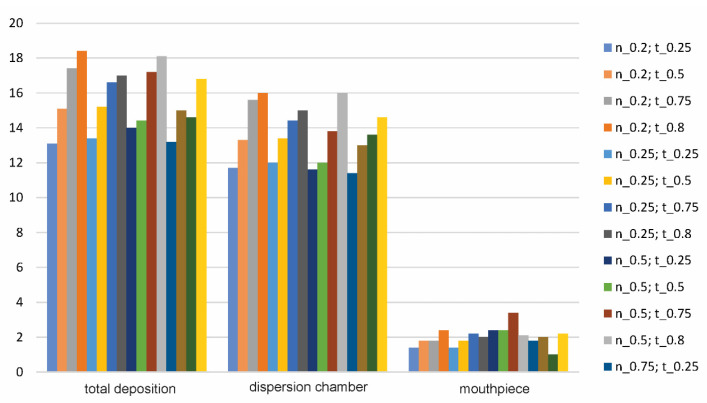
Percentage (%) of deposited particles for different variations of the tangential and normal COR values.

The percentage of deposited particles inside the inhaler, as well as the characteristic parts of the inhaler for different variations of the normal (nor.) and tangential (tan.) COR are shown in Figure 7. It should be noted that the dispersion chamber is defined as a capsule chamber + central chamber. 

Based on Figure 7, it can be observed that the highest percentage of deposited particles in the inhaler was obtained for nor. COR = 0.2 and tan. COR = 0.8, while the lowest percentage was obtained for the values of nor. COR = 0.2 and tan. COR = 0.25. It can also be seen that the combinations with a higher value of tan. COR and lower values of nor. COR gives higher percentages of deposited particles and vice versa. The percentage of deposited particles varies from 13.1% to 18.4% of the total number of injected particles. What is characteristic in numerical simulations for all COR values variations is that none of the deposited particles bounces back during airflow by rolling or sliding, i.e., all of the deposited particles remain stuck to the wall. This is mainly caused by a lower drag moment value than the adhesion moment value (condition for rolling), i.e., a lower drag force value than the adhesion friction value (condition for particle sliding). In addition, the wall friction velocity of the particles mainly has lower values for the critical wall shear velocities of rolling and sliding, which is also the reason why particles remain stuck to the wall.

**Figure 8:**
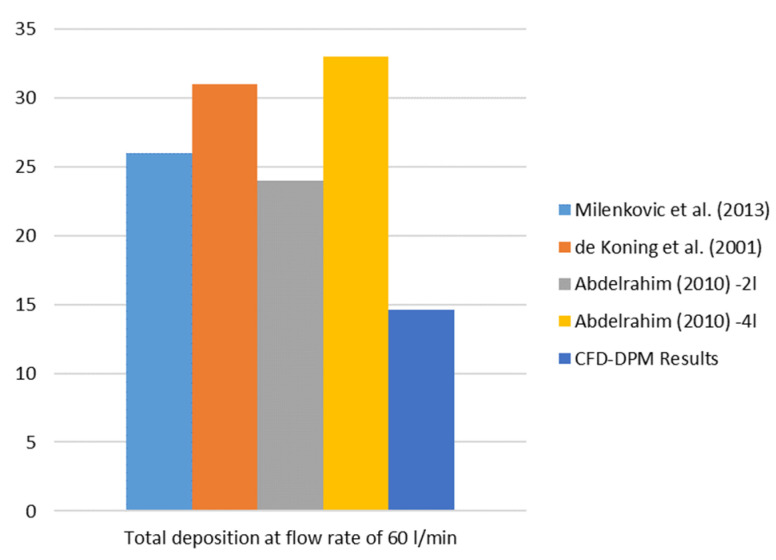
Comparison of the total particle deposition (%) for an airflow rate of 60 l/min.

A comparison of the results with the results from the in vitro studies in the literature [26,27], as well as the results from the numerical simulations presented in [13] in terms of total particle deposition, is shown in Figure 8. It should be emphasized that the comparison has been performed for other types of DPI devices (not the same as in our study) and different types of particles, with an airflow rate of 60 l/min. In addition, it is important to point out that the results of the numerical simulations (taken from literature for comparison) were obtained based on the same simplifications as are in this paper, i.e., neglection of inhaler humidity, temperature effect, etc. It can be concluded that the DPI device considered in this study has a lower percentage of total deposited particles (higher efficiency) than the devices from the literature at an airflow rate of 60 l/min. In addition, the in vitro results presented in [25] showed a 13–17% particle deposition for the same DPI formulation, meaning that the results from the CFD-DPM simulations correspond well to the in vitro results.

**Figure 9:**
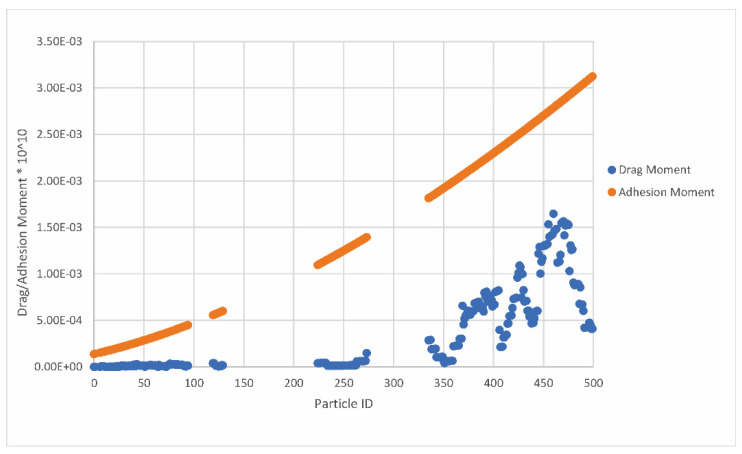
Relation between drag and adhesion moments for the same particle ID.

In relation to the definition of particle behavior as a result of drag and adhesion moment values, Figure 9 shows the drag and adhesion moment values for the same particle ID for COR_normal = 0.75 and COR_tangential = 0.75. 

**Figure 10:**
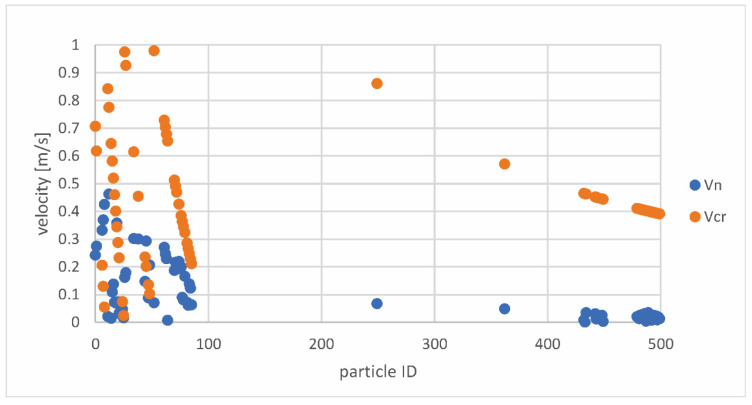
Relationship between the normal ($v_{n}$) and critical ($v_{cr}$) velocities for the same particle ID.

Based on Figure 9, it can be concluded that the drag moment is lower than the adhesion moment for all deposited particles; therefore, none of the particles will bounce back by rolling. The relationship between the normal and critical particle velocities for the same particle ID is shown in Figure 10.

Based on Figure 10, which was created based on the Stick.txt file, it can be seen that the particles written in this file will stick to the surface/inhaler wall since, according to theory, a particle that has a normal impact velocity smaller than the critical velocity will deposit in contact with the surface. This is consistent with the findings of Dahneke et al. [33], who determined that when the normal impact velocity drops, the relevance of the sticking force increases, resulting in lower rebound velocities. This indicates that there is no additional bouncing of the particles below a threshold amount of normal impact velocity, and the particles attach to the wall surface.

**Figure 11:**
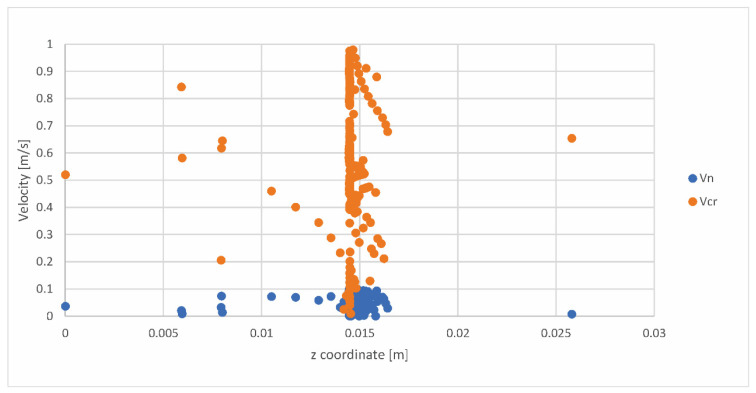
Dependence of critical ($v_{cr}$) and normal ($v_{n}$) particle velocities on z coordinate.

Figure 11 shows the dependence of the critical and normal particle velocities on the z coordinate.

Based on Figure 11**,** it can be concluded that most of the particles will bounce off of or stick to the grid zone, while in the lower part of the inhaler (dispersion chamber) and mouthpiece, a smaller number of particles impact the wall. The rest of the particles do not impact the wall on the path toward the outlet. This figure has been created based on the Impact.txt file.

**Figure 12:**
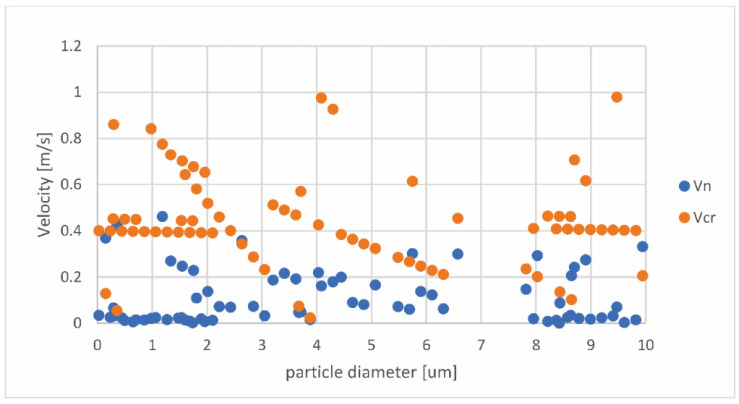
Dependence of critical ($v_{cr}$) and normal ($v_{n}$) velocities on the particle diameters.

Figure 12 shows the dependence of the critical and normal velocities on the particle diameters.

As can be seen in Figure 12, which was created based on the Stick.txt file, for the whole range of the investigated diameters, there are particles where $v_{n}<v_{cr}$. This means that these particles will stick to the inhaler’s wall. Additionally, this means that there is no specific range of particle diameters that will stick, but particles with any diameter size can be subjected to the sticking process.

**Figure 13:**
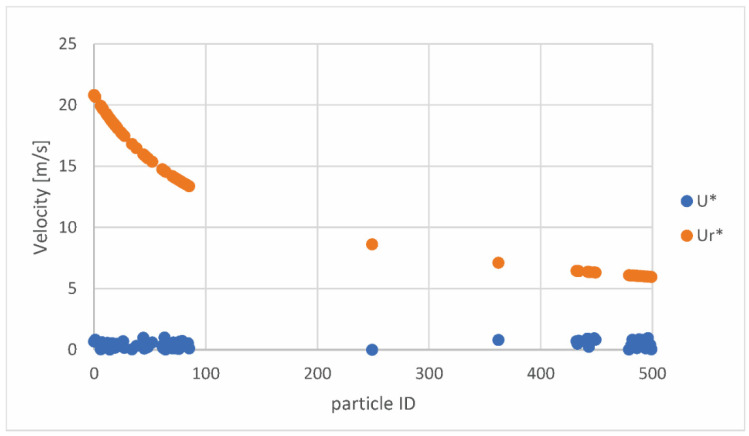
Dependence of wall shear velocity for rolling on particle ID.

Figure 13 shows the dependence of wall shear velocities for rolling on particle ID. 

It can be seen from Figure 13 that $u^{*}$ is always smaller than $u_{R}{}^{*}$. For a particle to detach by rolling or sliding, $u^{*}$ has to be greater than the critical wall shear velocities $u_{R}{}^{*}$ and $u_{S}{}^{*}$, respectively. As this condition is not fulfilled, the rolling mechanism is not present in the particle behavior inside the DPI. The same observation applies to the sliding mechanism. 

Regarding the limitations of this study, we need to note that a number of complex phenomena were not investigated, such as the effect of humidity inside the inhaler, the impact of temperature, etc. Additionally, the breakage of larger particles was not investigated, and this represents the basis for future investigation.

## 4. Conclusions

This study employed numerical simulations, particular computational fluid dynamics (CFDs), and a discrete phase model (DPM) to describe particle trajectories and behavior inside of a DPI. The methodology included a definition of the different mechanisms, including particle deposition and the detachment process. The impact of the particles on the DPI wall surface with a low normal velocity and low-impact angles is identified as the primary cause of particle deposition. Particles with reduced momentum before contact with a surface are less likely to bounce and, hence, come to a full stop. No detachment via sliding or rolling was observed as a mechanism, meaning that simple deposition and bouncing off are the primary behavior mechanisms of the particles. Differences in the coefficients of restitution (COR) did not have large effects on DPI efficiency. This study complements the results shown in our previous publication [25], where, in the former paper [25], the focus was on the comparative assessment of in silico and in vitro methods for the characterization of DPI aerodynamic performance, with emphasis on the in vitro results, while this study gives a more detailed explanation of the numerical modeling aspects, the forces acting on the particles, and the particle behavior mechanisms inside the DPI device. A comparison with the results from the literature for other DPI devices showed that a combination of the device and particles considered in this paper gives a lower percentage of deposited particles (higher efficiency) than the devices investigated in the literature. Future research will focus on expanding the mechanisms of particle behavior inside a DPI in terms of adding additional forces. Further investigation of different airflow rates and comparisons between the results of the numerical simulations and the in vitro experiments will be carried out in order to predict the particle deposition for another, nonsimulated, nonexperimentally investigated flow rate, meaning deposition trend prediction.

## Figures and Tables

**Table 1:** List of constants used to define particle sticking behavior.

Parameter Name	Symbol	Value	Unit	Reference
Young’s modulus for surface	$E_{s}$	4.1e^9^	Pa	[28]
Young’s modulus for particle	$E_{p}$	1e^9^	Pa	[28]
work of adhesion	$W_{A}$	0.039	J/m^2^	[9,32]
Poisson’s ratio for surface	$v_{s}$	0.35	/	[28]
Poisson’s ratio for particle	$v_{p}$	0.4	/	[28]
particle density	$\rho_{p}$	1230	kg/m^3^	[25]
air density (at 1013.25 hPa (abs) and 15 °C)	ρ	1.225	kg/m^3^	[28]
dynamic viscosity of fluid (air)	μ	1.7894e^−5^	N s/m^2^	[28]
correction factor for the near wall	f	1.7	/	[9,32]
Cunningham correction factor	$C_{u}$	1 (for spherical particles)	/	[9,32]
static coefficient of friction	$k_{s}$	0.5	/	[9,32]

**Table 2:** Total particle deposition (%) in the inhaler for different combinations of tangential and normal COR values.

		COR_Normal			
COR_tangential		0.20	0.25	0.50	0.75
	0.25	13.1	13.4	14.0	13.2
	0.50	15.1	15.2	14.4	15.0
	0.75	17.4	16.6	17.2	14.6
	0.80	18.4	17.0	18.1	16.8

## Data Availability

Not applicable.

## List of Used Symbols

**Symbol**	**Parameter Name**
V	Fluid velocity
u	Fluid velocity component parallel to the wall
$\upsilon_{n}$	Fluid velocity component normal to the wall
*p*	Pressure
υ	Kinematic viscosity
$\delta_{ij}$	Kronecker delta symbol
*k*	Specific turbulent energy
$F_{st}$	Sticking force
$F_{D}$	Drag force
$F_{L}$	Lift force
a	Deformation of the particle along the surface
$d_{p}$	Particle diameter
$v_{n}$	Normal (impact) velocity
$v_{cr}$	Critical (capture) velocity
$u^{*}$	Wall shear velocity
$u_{R}{}^{*}$	Wall shear velocity for rolling
$u_{s}{}^{*}$	Wall shear velocity for sliding
$d_{s}$	Distance of the first grid point from the wall
$C_{D}$	Drag coefficient
Rep	Reynolds coefficient
K_c_	Composite Young’s modulus
$E_{s}$	Young’s modulus for surface
$E_{p}$	Young’s modulus for particle
$W_{A}$	Work of adhesion
$v_{s}$	Poisson’s ratio for surface
$v_{p}$	Poisson’s ratio for particle
$\rho_{p}$	Particle density
$\rho_{M}$	Mixture density
ρ	Air density (at 1013.25 hPa (abs) and 15 °C)
μ	Dynamic viscosity of fluid (air)
f	Correction factor for the near wall
$C_{u}$	Cunningham correction factor
$k_{s}$	Static coefficient of friction
